# Supplementary material for: Stochastic Simulation of Biomolecular Networks in Dynamic Environments
Source: PLoS Comput Biol. 2016 Jun 1;12(6):e1004923. doi: 10.1371/journal.pcbi.1004923 (PMC4889045; doi:10.1371/journal.pcbi.1004923)
Supplement: S1 Text — Stochastic Simulation of Biomolecular Networks in Dynamic Environments. (PDF) [file pcbi.1004923.s002.pdf]

Supporting Information: Stochastic Simulation of Biomolecular Networks in Dynamic Environments

Margaritis Voliotis[1], Philipp Thomas[2], Ramon Grima[3,*] Clive G. Bowsher[1,†]
1 School of Mathematics, University of Bristol, U.K.
2 Department of Mathematics, Imperial College London, U.K.
3 School of Biological Sciences, University of Edinburgh, U.K.
∗ E-mail: ramon.grima@ed.ac.uk † E-mail: C.Bowsher@bristol.ac.uk,

The Extrande Simulation Approach

The collective firing times of a multivariate counting (or 'point') process, ignoring which channels fire, are called its **ground process**. One way to simulate a multivariate reaction counting process is to alternate between simulation of its ground process and allocation of firings of the ground process to the reaction channels. Here, we cannot simulate the ground process directly because of the stochastic, time-varying propensities between its firings. Our proof (see Appendix of main paper) relies on augmenting the reaction network with an extra, 'virtual' channel (giving the augmented system, Z), so as to make simulation of the ground process of the augmented system feasible, while ensuring that the simulated timings and types of biochemical reactions are unaffected by the firings of the extra channel.

The Extrande algorithm (Box 1) is in the class of multivariate point process thinning algorithms [31] and, in common with [32] makes use of a stochastic upper bound that is constant between events. In the Extrande method, the conditional propensity of the extra channel depends on the history of the extra channel (not just on the history of the original system, X), and so does the upper bound. The related Proposition in [32]omits the issue that the upper bound of its algorithm at t may not be measurable (computable) given the information $\{\mathcal{H}_t^X, \mathcal{I}\}$ alone (the joint history of X at time t, and [the σ-field generated by] the entire trajectory of any 'input', I). We therefore provide a new and more accessible proof. To see the dependence on the extra channel, note that the bound is in general updated in Step 3 of the Extrande algorithm (Box 1) after each each firing of the extra channel, as well as after biochemical reactions firing, hence the bound depends on the history of the extra channel.

Because the input I is exogenous (i.e., $\mathcal{I}$ is conditionally independent of $\mathcal{H}_t^Z$ given $\mathcal{H}_t^I$), its future trajectory can be made available during simulation of the biomolecular network to compute the bound, B. The look-ahead horizon, L, is also allowed to depend on $(\mathcal{H}_t^Z, \mathcal{I})$, with t the 'current time' in the algorithm, so L could, for example, adapt to past levels of thinning.

Implementations of Extrande

Frequently, the input process I will be given by a stochastic differential equation requiring numerical solution. Prior to applying Extrande, we then generate the trajectory of I on $[0, T]$ by first simulating values of I on a discrete grid (obtained, e.g., by the Euler-Maruyama method), with values for intermediate times obtained by a deterministic interpolation rule. The upper bound, B, in Step 3 (Box 1) is then chosen according to

$$B = \sup_{0 \le u < L} \sum_{j=1}^{M} a_j(X(t), I(t+u)), \tag{4}$$

where $a_j(X(t), I(t+u))$ gives the propensity of the j-th reaction channel at time $t+u$ *provided that no channel has fired since time t.* Once some channel $\{R_1, ..., R_{M+1}\}$ does fire, the bound B is reset according to Eq. 4 (with t the time of the most recent firing). We use this approach in Fig. 1 B&D.

Finding B according to Eq. 4 involves finding the supremum of I over the same interval, which can account for the majority of the CPU time needed to implement Extrande (see also Fig. 2). We also explored a second type of implementation of Extrande in which either: (a) the input $I(t+u) \le C$ with probability 1 for all $u < L$, for some (deterministic) constant, C; or (b) the input I is unbounded *ex ante* (that is, no such constant exists), but we can find an approximate ceiling, C, such that $Pr[I(t+u) \le C] = 1 - \epsilon$ for a small, predetermined ϵ and all $u < L$. In case (b), the Extrande algorithm is then applied using as input

the process $\tilde{I}(t) = \min\{I(t), C\}$. In both cases (a) and (b), the bound B in Step 3 is computed using the ceiling, C.

Examples of (a)—in which case Extrande is an exact simulation method unconditionally provided exact trajectories of the input can be simulated—are given in Fig. 1A and in computing the exact result shown in Fig. 4 below. Examples of (b) are given in Fig. 1C (where we set $\epsilon = 1.35 \times 10^{-3}$ and find C using the relevant quantile of the distribution of the underlying OU process) and Fig. 4. In Fig. 4, we demonstrate the convergence to the exact Extrande result of the case (b) implementation with approximate bound when ϵ is small. The advantage of the implementation in (b) is the potential for further large reductions in CPU time when (b) can be implemented but (a) cannot, by avoiding CPU time spent finding local ceilings of a pre-simulated input trajectory.

When the input I is a function of a univariate OU process, the transition density of the OU process is Gaussian and can be used to obtain $I(t)$ in Step 9 of the Extrande algorithm, avoiding pre-simulation of the OU trajectory on a fine grid when used in combination with case (b). We use this approach in Fig. 1C.

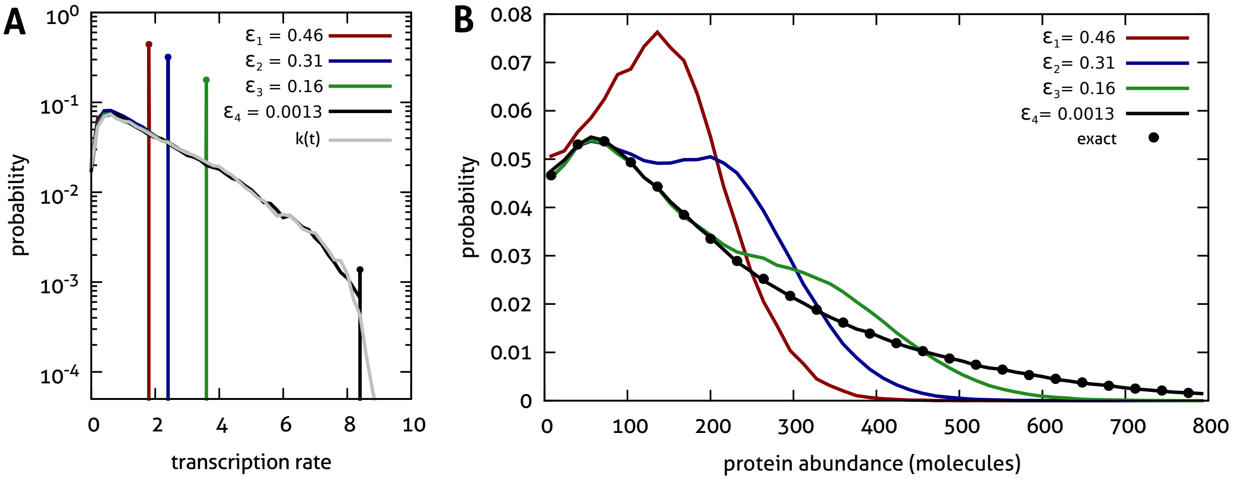

Figure A: **Convergence of Extrande method with approximate bound:** We verify the accuracy of the 'case (b)' implementation of Extrande for a fluctuating but saturating transcription rate given by $k(t) = 2K\exp(\xi(t))/(K + \exp(\xi(t)))$, with $K = 10$. Here $\xi(t)$ is a mean-zero Gaussian (OU) process with autocovariance $\langle \xi(t)\xi(t') \rangle = (5/4)e^{-\gamma|t-t'|}$, and $\gamma = 10^{-4}$. **(A)** compares the resulting distributions of the input, $\tilde{k}(t) = \min\{k(t), C(\epsilon)\}$, for different values of ϵ (using a bin size of 0.2). **(B)** We compare the distributions of protein numbers obtained for the gene expression model ($k_{\mathrm{dm}} = 4$, $k_{\mathrm{dp}} = 1$, $k_s = 100$) for decreasing values of ϵ. For large ϵ (red line), the tail of the distribution is substantially underestimated compared to the result from the exact, 'case (a)' Extrande algorithm (black diamonds). We find quantitative agreement with the exact Extrande result for $\epsilon = 1.35 \times 10^{-3}$ (black line). Decreasing the magnitude of fluctuations in the $k(t)$ helped to reduce the error in the protein distributions corresponding to larger ϵ (not shown). We speculate this is because intrinsic noise dominates protein fluctuations in the latter case.

Relation of Extrande with small look-ahead horizon, L, to the direct integral method

We write $a_j[X(t), I(t)]$ for the propensity of the jth reaction (conditional on $\{\mathcal{H}_t^X, \mathcal{I}\}$, for $j = 1, ..., M$, and $a_0(t) = \sum_{j=1}^M a_j[X(t), I(t)]$ for their sum. First, we analyse the behaviour of the Extrande method in the limit where the look-ahead horizon L is small and the input $I(t)$ can therefore be taken as constant over the horizon L. In this limit, $B \approx I(t)$ and therefore the extra, virtual channel never fires. We write $W_i = T_i - T_{i-1}$ for the waiting time to the firing of the ith reaction. In this limit, Extrande finds W_i by drawing a sequence of exponential random variables, $\{\mathcal{E}_k; k = 1, 2, ...\}$, with means $m(k) = [a_0(T_i + (k-1)L)]^{-1}$, and stopping the first time one of these random variables does not exceed L:

$$\Pr[W_i = (n-1)L + \mathcal{E}_n] = \Pr[\mathcal{E}_1 > L, \mathcal{E}_2 > L, ..., \mathcal{E}_{n-1} > L, \mathcal{E}_n \le L] = e^{-L\sum_{k=1}^{n-1} m(k)} - e^{-L\sum_{k=1}^{n} m(k)}, \quad (5)$$

for $n = 1, 2, ...$, which is the probability that n evaluations of a_0 in Step 3 of Extrande are used to draw W_i.

By a direct integral method we mean one that finds the waiting time, W_i, by numerical integration in

order to solve

$$\int_{t}^{t+W} \sum_{j=1}^{M} a_j(t+u)du = \mathrm{Exp}(1),$$

where Exp(1) denotes an exponential random variable with mean 1. The reaction occurrence time is then allocated to a reaction channel in the usual way for a direct method. Suppose the numerical integration uses a timestep equal to L. Then it is straightforward to show (again taking $I(t)$ constant over each small time-step of length L) that the probability that $W_i = (n-1)L$ is again given by the right-hand side of Eq. 5; this is the probability that n integration steps and hence n evaluations of a_0 are used to draw W_i.

We have shown that the Extrande method with small L and the direct integral method with step-size equal to that value of L give the same distribution for the number of evaluations of the propensity sum (number of iterations) used to draw each waiting time W_i, and therefore have the same expected CPU time for the propensity evaluations involved in drawing the sequence of W_i's. As $L \to 0$, the expected number of propensity evaluations diverges ($E[n] \to \infty$) for both methods, as does the expected CPU time for those propensity evaluations. The two methods are identical in the way they allocate reaction times to reaction channels. (The difference between them is that with small L, Extrande draws as many exponential random variables as evaluations of the propensity sum, n, for each waiting time (Eq. 5) whereas the direct integral method draws only one for each waiting time.)

The above analysis establishes that the expected CPU time of Extrande for some other choice of horizon, L^*, will be *below* the expected CPU time of the direct integral method (with small timestep L)—which includes the expected CPU time for propensity sum evaluation—whenever the expected CPU time for propensity evaluation in Step 3 (Box 1) for small L-Extrande is equal to (or exceeds) the *total* expected CPU time of Extrande using L^*. This is the case (to an adequate approximation) in Fig. 2A, setting, for example, $L^* = 10$h).

The Slow Input Approximation method

We present below the Slow Input Approximation (SIA) method used here for stochastic simulation over the interval $[0, T]$. The SIA method is based on Gillespie's direct method [14].

The reaction network has M reaction channels $\{R_1, ..., R_M\}$ with associated stoichiometries $\{v_1, ..., v_M\}$, and network state, X. The algorithm takes as input a function that simulates the dynamic input, I, and outputs the time t and state of the system whenever a reaction event occurs.

0. Initialise time $t \leftarrow 0$ and network state to $X \leftarrow X_0$.
1. Evaluate the reaction propensities $a_j(X, I(t))$ and their sum $a_0(t) = \sum_{j=1}^{M} a_j(X, I(t))$.
2. Draw independently an Exponential random variable, τ, with mean $1/a_0(t)$.
3. If $t + \tau > T$ end the algorithm; Else proceed to step 4.
4. Draw independently a Uniform$_{(0,1)}$ random variable, U; let j be the smallest positive integer less than or equal to M satisfying

$$\sum_{i=1}^{j} a_i(X, I(t)) \geq a_0(t)U.$$

5. Advance the time by replacing $t \leftarrow t + \tau$, evaluate $I(t)$ and update the system's state with the occurrence of reaction R_j at time t, $X \leftarrow X + \nu_j$.
6. Output (t, X) and return to step 1.

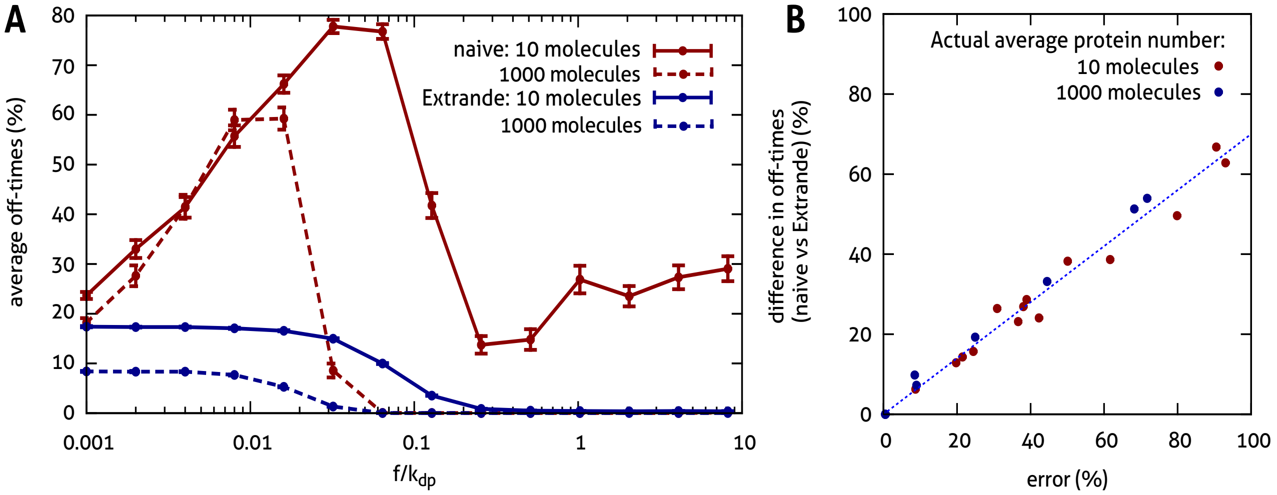

Figure B: **Failure of the Slow Input Approximation (SIA) method is mainly due to unphysical entrapment of mRNA and protein levels at zero copy number**: (A) The average fraction of time spent with both zero mRNA and zero protein molecules ('off-time') in a simulated 10 day trajectory, for the model of gene expression (Eq. 7) with circadian transcription rate $k(t) = 4(1 + \sin(2\pi f t)$ used in Fig. 1A (see main text). We observe that the SIA method (red) overestimates the off-time. The error bars have been estimated using the standard error of the mean of 100 trajectories. (B) The relative error of the SIA method (shown in Fig. 1A) is well-explained by the over-estimation of the off-time by the SIA method, compared to the Extrande estimate. The dashed blue line serves as a guide to the eye.

Relating to Figure 1

(**A**) Average protein number is based on $10,000$ realizations, $T = 1000$, and the integral was discretised using $\Delta t = 0.01$. Average protein numbers were varied via the translation rate, k_s, and the remaining parameters given by $k_{\mathrm{dm}} = 4$ and $k_{\mathrm{dp}} = 1$ (where the the unit of time is the inverse protein degradation rate). Median or mean protein degradation rates were estimated from proteomics data (*O. tauri* – $5h^{-1}$ [51], *S. crescentus* – $0.12h^{-1}$ [52], *S. cerevisiae* – $0.9h^{-1}$ [53], human cancer cells – $0.1h^{-1}$ [54], fibroblast – $0.01h^{-1}$ [55]).
(**B**) Average protein numbers were varied as above and the same parameter values. Autocorrelation times of the transcription rate, $k(t)$, were chosen according to typical cell cycle durations $t_{1/2} = \ln 2/\gamma$ from the literature (*O. tauri* – $24h$ [56], *S. cerevisiae* – $1.6h$, human cancer cells – $23h$ [54]). The average protein numbers were obtained from 100 independent realizations with $T = 10,000$ sampled every $\Delta t = 100$. The resulting 100 data points are then used to compute the error $|\langle n \rangle - \bar{n}_{\mathrm{ex}}|/\bar{n}_{\mathrm{ex}}$ (as obtained from Eq. (1) of the main text in the stationary case) and the sampling error given by one standard deviation of the bootstrap distribution.
(**C**) & (**D**) Parameters used are $f = (1/24)h^{-1}$, $k_{\mathrm{dp}} = 5h^{-1}$, $k_{dm} = 20h^{-1}$, $k_s = 500h^{-1}$.

In order to understand better the source of the error of the SIA method shown in Fig. 1A, we considered the fraction of the 10 day simulated trajectories during which both mRNA and protein levels are zero (the 'off-time'), comparing the SIA result to the one obtained using Extrande (Fig. B). The relative error of the SIA method is well-explained by the over-estimation of the off-time by the SIA method. We have verified, throughout Fig. 1A close agreement of the results generated using Extrande with the corresponding analytical results (Fig. C).

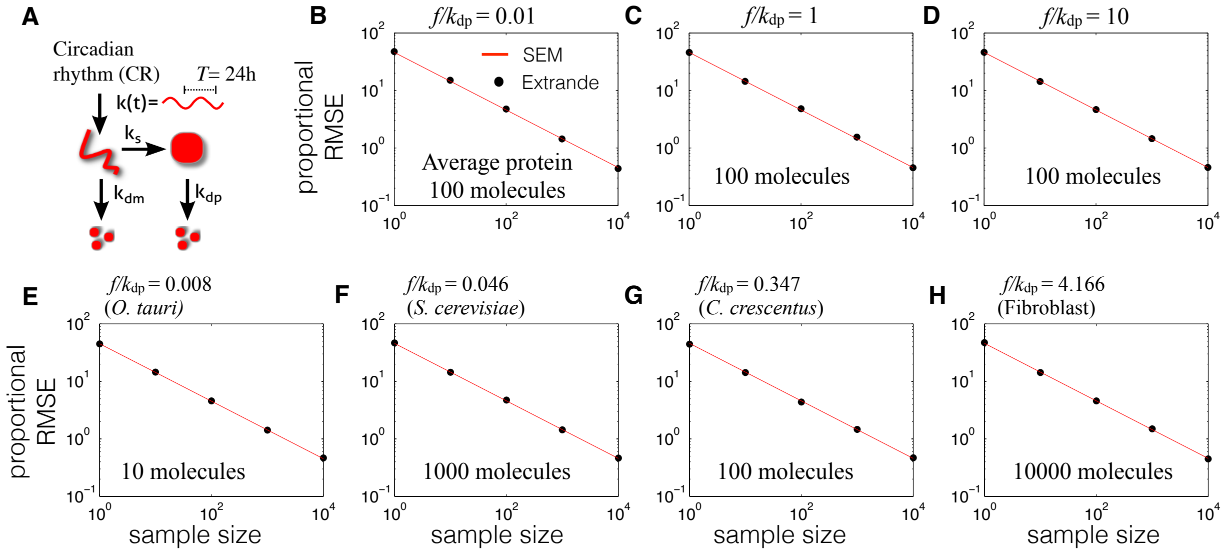

Figure C: **Accuracy of the Extrande method.** (A) We use Extrande to sample trajectories of protein levels from the model of gene expression used in Fig. 1A [Eq. 7 with circadian transcription rate, $k(t) = 4(1+sin(2\pi ft))$, $f^{-1} = 24$h], for a range of protein degradation rates ($k_{\rm dp}$) and average protein levels (10-10000 molecules). (B-H) We calculate the proportional root mean square error (RMSE) between the (sample) average protein number and the exact, time-dependent solution of the model (derived as in [10] from datasets with different sample sizes. In all cases as the sample size is increased the proportional RMSE diminishes in agreement with the relative standard error of the mean (SEM) obtained from the model. We vary the average protein numbers using the translation rate (k_s) and set $k_{\rm dm} = 4k_{\rm dp}$.

Relating to Figure 2

Figure 7 analyses further the performance of the Extrande and MN integral methods using the SynDM network (see Fig. 3), with an OU input process. Note the conspicuous relative error (> 60 %) of the MN integral method using an integration time-step of 1s. We were unable to report the relative error for integration time-steps $< 10^{-4}$h owing to the CPU times needed to obtain averages across 1000 cells.

Relating to Figure 3

(**A**) Table 1 lists the reactions and parameter values used in our models of the competence modules of the wild-type *B. subtilis* and the Synthetic Decision-Making (SynDM) networks. The wild-type module is based on the stochastic model presented in [57]; here, the ComK and ComS promoters are modelled using mass-action reactions kinetics (rather than Hill functions) and a group of rate constants is rescaled in order to ensure that the model yields a fraction of competent cells (17%) and an average time of entering competence (12h) in rough agreement with those reported in the literature [45, 46] (when pComA is constant at 1000 molecules). Table 2 lists the reactions and parameter values used in our model of the upstream signaling module determining the dynamics of pComA. The model is based on the descriptions of the signaling given in [58–60] and parameterised using biophysically realistic parameter values.

(**B-G**) ComK is the master transcription factor that drives gene expression and the physiological changes required for competence [46] (migration of ComGA and other proteins to the cell poles, assembly of DNA uptake and processing machinery at the poles, etc.). We therefore prefer not to model entry into competence as occurring the first time the ComK level exceeds a threshold, but to use a different model of ComK-driven progress and entry into functional competence:

$$\text{Progress}(t) = k \int_0^t \text{ComK}(s) ds, \qquad (6)$$

where k is an effective rate of ComK-driven differentiation. A cell is taken to enter (functional) competence at the time when Progress$(t) = 1$. The value of the parameter k is set so that the wild-type and SynDM

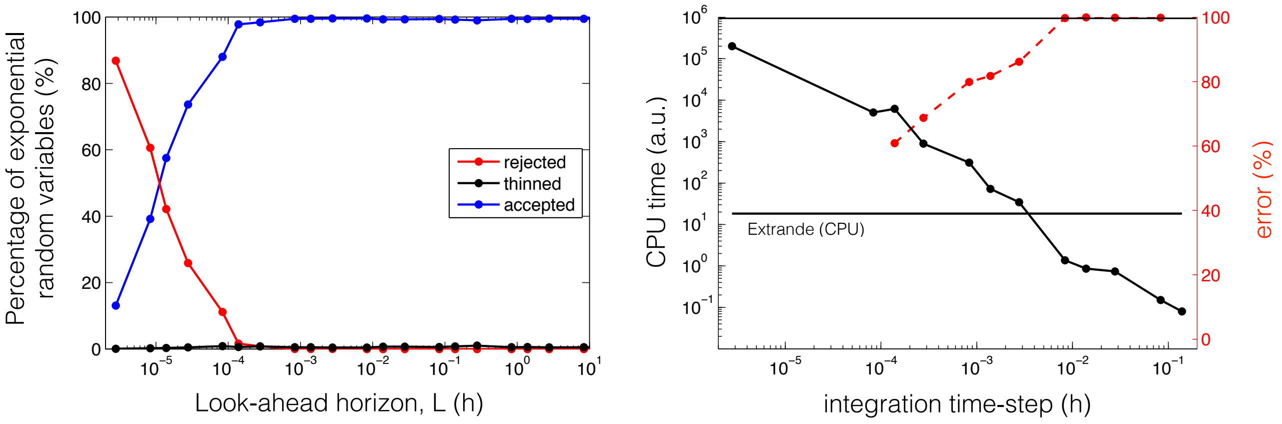

Figure D: **Comparison of the Extrande and integral method using the SynDM network.** The SynDM network is as in Fig. 3 and we use a Gaussian OU input process to model pComA fluctuations (mean= 1000 molecules, CV= 0.5, lifetime= 1h). **(A)** Percentages of exponential random variables generated in Step 4 of Extrande (Box 1) that are rejected, thinned, and accepted, as a function of the look-ahead horizon, L. Data shown is taken from simulation of a single SynDM cell over 1h of calendar time. **(B)** CPU times and percentage errors for the Modified Next (MN) integral method as a function of the integration time-step. The error is the absolute relative error in the (conditional) average of the 'Progress to competence' variable at 1h (see Eq. 8), given a particular pComA input trajectory; the error is calculated relative to the value obtained using Extrande. For both Extrande and the MN integral method, the average Progress to competence is calculated over 1000 independent cells. CPU time corresponds to simulation of a single cell over 1 h of calendar time, and for Extrande corresponds to an intermediate value of L (see Fig. 2C).

networks give equal fractions of competent cells with a constant level of pComA (1000 molecules).

To study the effect of extrinsic fluctuations from upstream signaling on the competence decision we simulated the wild-type and SynDM networks using Gaussian, Ornstein-Uhlenbeck (OU) input processes to model pComA fluctuations. To obtain the parameters of the OU process we first apply the linear noise approximation (LNA) [43] to the upstream signaling module in Table 2 to find the mean (1000 molecules), CV (0.35) and autocorrelation function (ACF) of steady-state pComA levels. We find that the autocorrelation function (ACF) of pComA is well fitted by a single exponential function. Therefore we use a single OU process (with mean and variance obtained from the LNA, and lifetime obtained from the fit) to describe pComA fluctuations. We verified the adequacy of our single-OU approximation by checking that modeling the pComA input as a sum of two independent OU processes yields quantitatively similar fractions of competent cells (this yielded 0.39 cf 0.40 for SynDM, and 0.17 cf 0.17 for the wild-type). We obtain the other OU input process specifications examined by keeping the mean constant ($\mu = 1000$) and varying the lifetime (τ) and variance (σ^2) of the process. OU input trajectories used were presimulated using time-step of 1s.

To confirm the importance of modeling synthesis and degradation of the signaling proteins in the upstream signal transduction module, we repeated the LNA analysis using a variant of the signaling module in which ComA, ComP and RapC species were kept constant at their average levels (by removing all corresponding gene expression and degradation reactions from the model). This formulation of the model yielded an OU process with the same mean (1000 molecules), but with greatly reduced CV (0.07) and autocorrelation time (28 sec). All fractions reported in panels (E-G) were based on 1000 independent cells.
(**H**) The autocorrelation function of pComA that was obtained from the LNA analysis of the signaling module was fitted to:

1. the ACF of an OU process, $\exp(-t/\tau)$, yielding $\tau = 5$h; and
2. the ACF of the sum of two independent OU processes, $p \cdot \exp(-t/\tau_1) + (1-p) \cdot \exp(-t/\tau_2)$, yielding $\tau_1 = 5.4$h, $\tau_2 = 6.0 \cdot 10^{-3}$h, and $p = 0.941$.

Least-squares fits were performed in Matlab using the *fit* function.

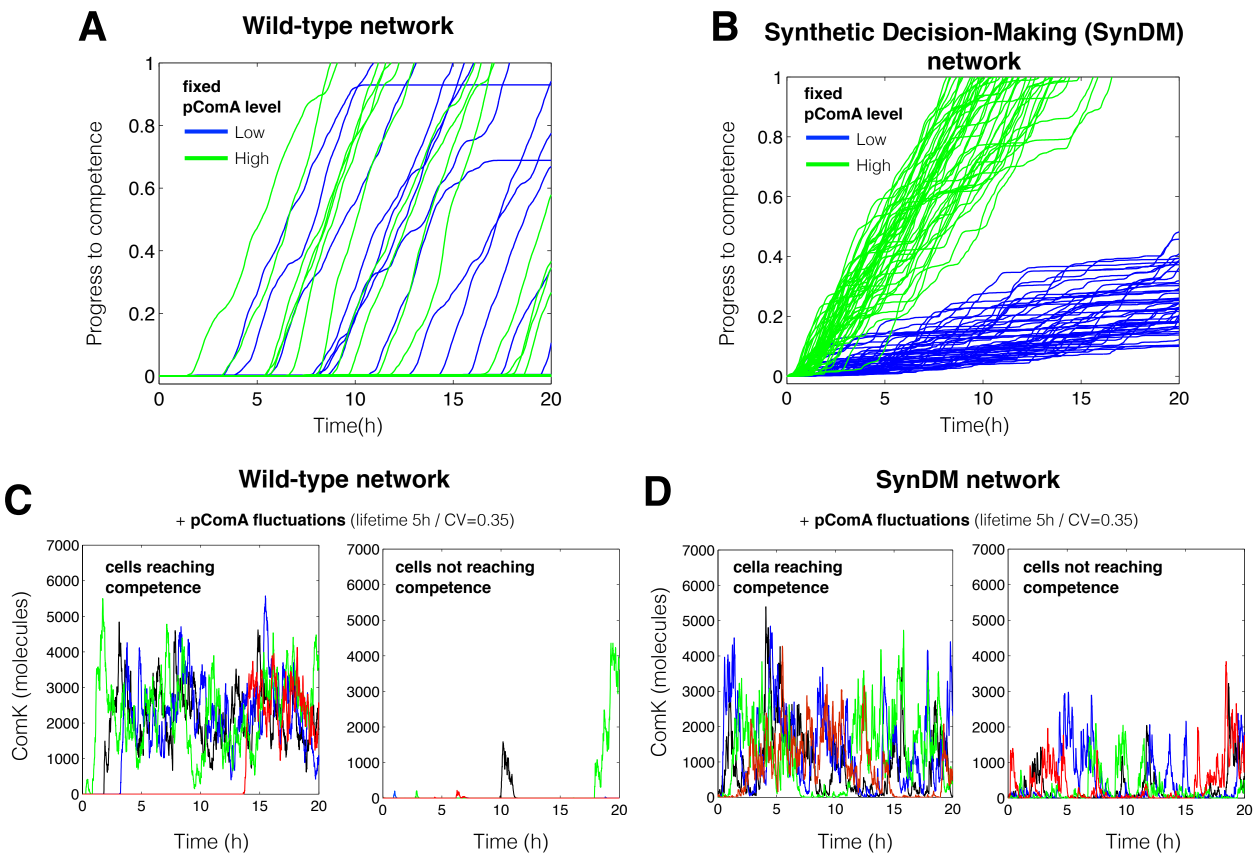

Figure E: **Progress to competence for cells containing the wild-type and SynDM network.** (**A**–**B**) Time courses of Progress to competence for 50 cells, when pComA levels are constant across cells and over time, shown at a low (650 molecules, blue) and a high (1350 molecules, green) pComA level. (**C**) Selected ComK trajectories for wild-type cells that either enter competence (left) or not (right). Each cell has an independent Gaussian, OU input process (mean=1000, CV=0.35, lifetime 5h) derived from LNA anlaysis of the upstream signalling module, as in Fig. 3B. (**D**) The analogue of C for cells containing the SynDM network, as in Fig. 3D.

Model of inter-cellular quorum sensing. We assume a population of N identical cells, each with a copy of the upstream quorum signaling module listed in Table 2. Communication between cells is achieved via the molecular species CSF and ComX, which are produced by cells and released to the extracellular space with volume V_e. The macroscopic dynamics of the extracellular concentration of these two species are governed by the following equations:

$$\frac{d[\mathrm{CSF}_e]}{dt} = \frac{1}{V_e}\left\{\sum_{i=1}^{N} V_c k_{21}[\mathrm{CSF}]_i - V_c k_{-21}[\mathrm{CSF}_e]\right\} - k_{20}[\mathrm{CSF}_e],$$

$$\frac{d[\mathrm{ComX}_e]}{dt} = \frac{1}{V_e}\{\sum_{i=1}^{N} V_c k_{-10}[\mathrm{ComP\text{-}ComX}]_i - V_c^2 k_{10}[\mathrm{ComP}]_i[\mathrm{ComX}_e] + $$
$$+ V_c k_{-11}[\mathrm{pComP\text{-}ComX}]_i - V_c^2 k_{11}[\mathrm{pComP}]_i[\mathrm{ComX}_e] + V_c k_{18}[\mathrm{ComX}]_i\} - k_{17}[\mathrm{ComX}_e],$$

where index i denotes the concentration of the intracellular species in the ith cell (having volume V_c). Since the cells are identical, the sum simplifies and we can rescale the appropriate rates by N/V_e. Then we solve the resulting ODE system (describing the macroscopic dynamics of one cell and the two extracellular species) to obtain the steady-state levels of all species in the signaling module. The LNA is then applied to the model in Table 2, treating the levels of $[\mathrm{CSF}_e]$ and $[\mathrm{ComX}_e]$ as constants equal to their steady-state values and absorbing them into the appropriate rate constants (and ignoring the now redundant $[\mathrm{CSF}_e]$ and $[\mathrm{ComX}_e]$ species of the LNA). The approach assumes that fluctuations in the extracellular species are sufficiently rapid that their effect on each cell is negligible.

References

[51] Martin SF, Munagapati VS, Salvo-Chirnside E, Kerr LE, Le Bihan T. Proteome turnover in the green alga Ostreococcus tauri by time course 15N metabolic labeling mass spectrometry. J Proteome Res. 2011;11:476–486.

[52] Chabot JR, Pedraza JM, Luitel P, van Oudenaarden A. Stochastic gene expression out-of-steady-state in the cyanobacterial circadian clock. Nature. 2007;450:1249–1252.

[53] Belle A, Tanay A, Bitincka L, Shamir R, O'Shea EK. Quantification of protein half-lives in the budding yeast proteome. Proc Natl Acad Sci USA. 2006;103:13004–13009.

[54] Eden E, Geva-Zatorsky N, Issaeva I, Cohen A, Dekel E, Danon T, et al. Proteome half-life dynamics in living human cells. Science. 2011;331:764–768.

[55] Schwanhäusser B, Busse D, Li N, Dittmar G, Schuchhardt J, Wolf J, et al. Global quantification of mammalian gene expression control. Nature. 2011;473:337–342.

[56] Farinas B, Mary C, de O Manes CL, Bhaud Y, Peaucellier G, Moreau H. Natural synchronisation for the study of cell division in the green unicellular alga Ostreococcus tauri. Plant Mol Biol. 2006;60:277–292.

[57] Espinar L, Dies M, Cagatay T, Süel GM, Garcia-Ojalvo J. Circuit-level input integration in bacterial gene regulation. Proc Natl Acad Sci USA. 2013;110:7091–7096.

[58] Solomon JM, Magnuson R, Srivastava A, Grossman AD. Convergent sensing pathways mediate response to two extracellular competence factors in Bacillus subtilis. Genes Dev. 1995;9:547–558.

[59] Lazazzera BA, Kurtser I, McQuade RS, Grossman AD. An autoregulatory circuit affecting peptide signaling in Bacillus subtilis. J Bacteriol. 1999;181:5193–5200.

[60] Schultz D, Wolynes PG, Jacob EB, Onuchic JN. Deciding fate in adverse times: sporulation and competence in Bacillus subtilis. Proc Natl Acad Sci USA. 2009;106:21027–21034.

No.	Reaction	Rate constants	Values
K = ComK, S = ComS, P_K indicates ComK Promoter, P_S indicates ComS Promoter			
**Wild-type network**			
1	MecA + K $\rightleftharpoons$ MecA-K	$k_1(\rightarrow), k_{-1}(\leftarrow)$	$k_1 = 5.1 \cdot 10^{-6}, k_{-1} = 2.5 \cdot 10^{-3}$
2	MecA-K $\rightarrow$ MecA	k_2	$k_2 = 0.125$
3	P_K + K $\rightleftharpoons$ P_K-K	$k_3(\rightarrow), k_{-3}(\leftarrow)$	$k_3 = 10^{-4}, k_{-3} = 0.5$
4	$P_K \rightarrow P_K$ + mK	k_4	$k_4 = 3.125 \cdot 10^{-5}$
5	P_K-K $\rightarrow$ P_K-K + mK	k_5	$k_5 = 0.781$
6	mK $\rightarrow$ mK + K	k_6	$k_6 = 1$
7	MecA + S $\rightleftharpoons$ MecA-S	$k_7(\rightarrow), k_{-7}(\leftarrow)$	$k_7 = 1.75 \cdot 10^{-5}, k_{-7} = 2.5 \cdot 10^{-4}$
8	MecA-S $\rightarrow$ MecA	k_8	$k_8 = 7 \cdot 10^{-4}$
9	P_S + pComA $\rightleftharpoons$ P_S-pComA	$k_9(\rightarrow), k_{-9}(\leftarrow)$	$k_9 = 1.546 \cdot 10^{-4}, k_{-9} = 0.5$
10	P_S + K $\rightleftharpoons$ P_S-K	$k_{10}(\rightarrow), k_{-10}(\leftarrow)$	$k_{10} = 3.201 \cdot 10^{-4}, k_{-10} = 0.5$
11	$P_S \rightarrow P_S$ + mS	k_{11}	$k_{11} = 0$
12	P_S-pComA $\rightarrow$ P_S-pComA + mS	k_{12}	$k_{12} = 0.0125$
13	P_S-K $\rightarrow$ P_S-K + mS	k_{13}	$k_{13} = 0$
14	mS $\rightarrow$ mS + S	k_{14}	$k_{14} = 1$
15	mK $\rightarrow \emptyset$	k_{15}	$k_{15} = 0.025$
16	mS $\rightarrow \emptyset$	k_{16}	$k_{16} = 0.025$
**Synthetic Decision-Making (SynDM) network**<br>Reactions 1-4, and 6-16 included as in Wild-type			
17	P_K + pComA $\rightleftharpoons$ P_K-pComA	$k_{17}(\rightarrow), k_{-17}(\leftarrow)$	$k_{17} = 2 \cdot 10^{-5}, k_{-17} = 0.5$
18	P_K-K + pComA $\rightleftharpoons$ P_K-K-pComA	$k_{18}(\rightarrow), k_{-18}(\leftarrow)$	$k_{18} = 2 \cdot 10^{-5}, k_{-18} = 0.05$
19	P_K-pComA + ComK $\rightleftharpoons$ P_K-K-pComA	$k_{19}(\rightarrow), k_{-19}(\leftarrow)$	$k_{19} = 10^{-4}, k_{-19} = 0.05$
20	P_K-K $\rightarrow$ P_K-K + mK	k_{16}	$k_{20} = 0.375$
21	P_K-ComA $\rightarrow$ P_K-pComA + mK	k_{21}	$k_{21} = 0.1$
22	P_K-K-pComA $\rightarrow$ P_K-K-pComA + mK	k_{22}	$k_{22} = 0.781$
**Initial conditions** (Wild-type & SynDM network)			
MecA		1000 molecules/cell	
P_S		1 molecule/cell	
P_K		1 molecule/cell	
All other species are initialised at 0 molecules/cell			

Table 1: **Reactions and parameter values for the competence modules in the *B. subtilis* wild-type and the Synthetic Decision-Making networks**. All reactions are formulated in terms of stochastic mass action kinetics. First order rate constants are given in units of [molecules sec]$^{-1}$, and second order rate constants in [molecules]$^{-2}$[sec]$^{-1}$.

No	Reaction	Rate constants	Values
**Upstream quorum signaling module (Wild-type and SynDM networks)**			
1	pComP + ComA $\rightarrow$ ComP+pComA	k_1	k_1=0.1
2	pComP-ComX + ComA $\rightarrow$ ComP-ComX+pComA	k_2	$k_2 = 0.1$
3	RapC + pComA $\rightleftharpoons$ RapC-pComA	$k_3(\rightarrow), k_{-3}(\leftarrow)$	$k_3 = 0.001, k_{-3} = 0.1$
4	RapC-pComA $\rightarrow$ RapC + pComA	k_4	$k_4 = 0.5$
5	ComA $\rightarrow \emptyset$	k_5	$k_5 = 5.5 \cdot 10^{-5}$
6	mRN$A_{ComA} \rightarrow \emptyset$	k_6	$k_6 = 0.001$
7	$P_{ComA} \rightarrow P_{ComA}$ + mRNA$_{ComA}$	k_7	$k_7 = 0.01$
8	mRN$A_{ComA} \rightarrow$ mRNA_{ComA} + ComA	k_8	$k_8 = 0.025$
9	ComP-ComX $\rightarrow$ pCom-ComX	k_9	$k_9 = 1$
10	ComP + Com$X_e \rightleftharpoons$ ComP-ComX	$k_{10}(\rightarrow), k_{-10}(\leftarrow)$	$k_{10} = 0.00233, k_{-10} = 0.1$
11	pComP + ComX$_e \rightleftharpoons$ pComP-ComX	$k_{11}(\rightarrow), k_{-11}(\leftarrow)$	$k_{11} = 0.00233, k_{-11} = 0.1$
12	ComP,pComP,pComP-ComX,ComP-ComX $\rightarrow \emptyset$	k_{12}	$k_{12} = 5.5 \cdot 10^{-5}$
13	mRN$A_{ComP} \rightarrow \emptyset$	k_{13}	$k_{13} = 0.001$
14	$P_{ComP} \rightarrow P_{ComP}$ + mRNA$_{ComP}$	k_{14}	$k_{14} = 0.01$
15	mRN$A_{ComP} \rightarrow$ mRNA_{ComP} + ComP	k_{15}	$k_{15} = 0.001$
16	ComX $\rightarrow \emptyset$	k_{16}	$k_{16} = 5.5 \cdot 10^{-5}$
17	Com$X_e \rightarrow \emptyset$	k_{17}	$k_{17} = 10$
18	ComX $\rightarrow$ ComX$_e$	k_{18}	$k_{18} = 100$
19	$\emptyset \rightarrow$ ComX	k_{19}	$k_{19} = 50$
20	CS$F_e \rightarrow \emptyset$	k_{20}	$k_{20} = 10$
21	CSF $\rightleftharpoons$ CSF$_e$	$k_{21}(\rightarrow), k_{-21}(\leftarrow)$	$k_{21} = 0.1, k_{-21} = 0.1$
22	PhrC $\rightarrow$ CSF	k_{22}	$k_{22} = 100$
23	PhrC $\rightarrow \emptyset$	k_{23}	$k_{23} = 5.5 \cdot 10^{-5}$
24	mRN$A_{PhrC} \rightarrow \emptyset$	k_{24}	$k_{24} = 0.005$
25	$P_{PhrC} \rightarrow P_{PhrC}$ + mRNA_{PhrC}	k_{25}	$k_{25} = 0.1$
26	mRN$A_{PhrC} \rightarrow$ mRNA_{PhrC} + PhrC	k_{26}	$k_{26} = 0.0025$
27	RapC + CSF $\rightleftharpoons$ Rap$C_{inactive}$	$k_{27}(\rightarrow), k_{27}(\leftarrow)$	$k_{27} = 0.001, k_{27} = 0.1$
28	RapC,Rap$C_{inactive} \rightarrow \emptyset$	k_{28}	$k_{28} = 5.5 \cdot 10^{-5}$
29	mRN$A_{RapC} \rightarrow \emptyset$	k_{29}	$k_{29} = 0.001$
30	$P_{RapC} \rightarrow P_{RapC}$ + mRNA_{RapC}	k_{30}	$k_{30} = 0.01$
31	mRN$A_{RapC} \rightarrow$ mRNA_{RapC} + RapC	k_{31}	$k_{31} = 0.001$

Table 2: **Reactions and parameter values for the upstream signaling module determining the dynamics of pComA, in both the Wild-type and SynDM networks**. The quorum sensing molecules transported into the extracellular space (e) are ComX and CSF. CSF is also imported into the cell. ComP is a histidine kinase receptor that binds the ligand ComX, and ComP-ComA together form a two component phosphorelay mechanism. RapC dephosphorylates pComA, and is itself sequestered by CSF inside the cell. The model is based on the descriptions of the upstream signaling given in [58–60] and parameterised using biophysically realistic parameter values. Notice the inclusion here of synthesis and degradation reactions for the signaling proteins ComA, ComP, ComX and PhrC/CSF. All reactions are formulated in terms of stochastic mass action kinetics. First order rate constants are given in units of [molecules sec]$^{-1}$, and second order rate constants in [molecules]$^{-2}$[sec]$^{-1}$. The ratio of the intracellular to extracellular volume was set to 500.
